# Correlation between gene expression levels under drought stress and synonymous codon usage in rice plant by in-silico study

**DOI:** 10.1371/journal.pone.0237334

**Published:** 2020-08-10

**Authors:** Fatemeh Chamani Mohasses, Mahmood Solouki, Behzad Ghareyazie, Leila Fahmideh, Motahhareh Mohsenpour

**Affiliations:** 1 Department of Plant Breeding and Biotechnology (PBB), Faculty of Agriculture, University of Zabol, Zabol, Iran; 2 Agricultural Biotechnology Research Institute of Iran (ABRII), Agricultural Research Education and Extension Organization (AREEO), Karaj, Iran; Louisiana State University, UNITED STATES

## Abstract

We studied the correlation of synonymous codon usage (SCU) on gene expression levels under drought stress in rice. Sixty genes related to drought stress (with high, intermediate and low expression) were selected from rice meta-analysis data and various codon usage indices such as the effective number of codon usage (ENC), codon adaptation index (CAI) and relative synonymous codon usage (RSCU) were calculated. We found that in genes highly expressing under drought 1) GC content was higher, 2) ENC value was lower, 3) the preferred codons of some amino acids changed and 4) the RSCU ratio of GC-end codons relative to AT-end codons for 18 amino acids increased significantly compared with those in other genes. We introduce ARSCU as the Average ratio of RSCUs of GC-end codons to AT-end codons in each gene that could significantly separate high-expression genes under drought from low-expression genes. ARSCU is calculated using the program ARSCU-Calculator developed by our group to help predicting expression level of rice genes under drought. An index above ARSCU threshold is expected to indicate that the gene under study may belong to the “high expression group under drought”. This information may be applied for codon optimization of genes for rice genetic engineering. To validate these findings, we further used 60 other genes (randomly selected subset of 43233 genes studied for their response to drought stress). ARSCU value was able to predict the level of expression at 88.33% of the cases. Using third set of 60 genes selected amongst high expressing genes not related to drought, only 31.65% of the genes showed ARSCU value of higher than the set threshold. This indicates that the phenomenon we described in this report may be unique for drought related genes. To justify the observed correlation between CUB and high expressing genes under drought, possible role of tRNA post transcriptional modification and tRFs was hypothesized as possible underlying biological mechanism.

## Introduction

Abiotic stresses such as drought, salinity, low or high temperatures and other environmental extremes negatively affect crop growth and reduce crop yield on a global scale [1]. Among these stresses, drought stress is the most important factor-limiting yield in agricultural systems in arid and semi-arid regions [2]. Rice (*Oryza sativa*), a well-known cost-effective cereal in the world, is very sensitive to drought stress because of its limited adaptation to water-deficit conditions. Although rice germplasm has a large amount of functional genetic diversity for drought tolerance–related traits/mechanisms [3], breeding for drought tolerance is still a challenge for breeders because of its complex genetic nature with higher environmental plasticity and multiple metabolic pathways involved. There are hopes that transgenic technology can improve stress tolerance by introducing novel exogenous genes or altering the expression levels of endogenous genes [4]. Researchers have endeavored during recent decades to generate transgenic crops with improved tolerance against abiotic stresses. The first step of transgenic research is the identification of genes serving as key regulators of different metabolic pathways, including osmolyte synthesis, ion homeostasis through selective ion uptake, antioxidant defense system and other frontline defense pathways [5]. Rice is currently considered a model crop for genetic engineering with well-developed tissue culture and gene transfer protocols. There are numerous reports on the production of genetically modified (GM) rice for such traits as enhanced resistance against insect pests and/or herbicide tolerance [6–10]. In some cases, approvals have even been issued for such GM rice plants in Japan, Canada, Colombia, Mexico, Honduras, New Zealand, Philippines and the United States [11]. However, unlike in the case of drought tolerant maize (DroughtGard) [12], to date there is no verifiable report of production and/or approval of any GM drought-tolerant rice. Despite the ever-increasing discovery and introduction of genes affecting different traits, the challenge remains to discover and explore genes effectively rendering abiotic stress tolerance [13]. Plant species are reported to indicate wide diversity in terms of their gene expression, physiology and stress response under different environmental conditions [14]. A key to progress towards breeding better crops for stressed conditions is to understand the alterations in cellular, biochemical and molecular machinery that occur in response to stress [15]. A particular stress changes the expression level of specific genes in a species-dependent fashion. It causes differences in the efficiency of signal perception and subsequent transcriptional changes leading to elicitation of a specific response and adaptation and finally enhanced stress tolerance [16]. In addition, gene expression levels correlate with several features of the underlying genes and encoded proteins, including synonymous codon (codons encoding the same amino acid) usage, amino acid composition, rates of protein evolution, and the length of coding sequence [17,18]. Synonymous codons are not equally present in all genes and/or species. This phenomenon, called codon usage bias (CUB), is ubiquitous in all organisms. Mutational pressure and natural selection are considered the two major factors contributing to CUB [19]. The correlation of CUB with gene expression levels has been reported in organisms from all domains of life [20–29]. Codon usage presents the specific pattern of gene expression and it has been noted that the use of the same codon is preferred in gene expression under the same physiological state [30]. It has been shown that the overall preference of amino acid usages and codon usage preferences in the proteins of a given organism were significantly affected by major environmental factors [31]. This study notes the presence of a correlation between cellular function and codon usage profiles of the genes in the studied pairs. In addition, some previous studies showed the existence of correlation between gene expression levels and CUB [18,32,33]. Furthermore, several published studies support the idea that translation of specific codon-biased transcripts in stress response genes can be regulated by tRNA modifications [34–36]. Wobble-base tRNA modification levels have the potential to work in concert with codon usage patterns in specific transcripts to regulate translation of response proteins. Dedon et al. [37] designated these as modification tunable transcripts (MoTTs). Modified tRNA directly participate in translation. However, unmodified mature tRNAs or pre-tRNAs are processed into various types of transfer RNA-derived fragments known as tRFs [38–41]. Production of tRFs is promoted by abiotic stress conditions to eventually control gene expression by transcriptional, post-transcriptional and translational regulation [42].

Hence, knowledge of the codon usage and codon-pair context patterns of plants and underlying evolutionary forces will be useful to understand the molecular mechanism of environmental adaptation and biological diversity of each species [43]. In addition, this information can be applied for codon optimizations of diverse sets of genes to be used in plant transformation programs. Here, we use genome sequence and genome-wide expression data of drought responsive genes to investigate the relationship between gene expression levels and CUB in rice.

## Material and methods

### Selection of genes with different expression levels under drought stress

Sixty genes associated with drought stress in rice were identified through a structured literature survey from meta-analysis data [44–46]. Then the genes were divided based on their expression levels under drought condition into three categories: high-, intermediate- and low-expression genes. Complete coding DNA sequences of these genes were accessed from the NCBI nucleotide database [47]. In the present study, high-expression genes included NAC transcription factors, zinc finger proteins, AP2/ERF transcription factors, LEA proteins, phosphatidylethanolamine binding proteins, peptide transporters, dioxygenases, actin-binding proteins, protein phosphatases, dehydrins and membrane proteins family. These genes have been reported to show high expression under drought stress in different experiments (Table 1). In addition to the high expression, some of these genes showed enhanced drought tolerance in reported genetic engineering experiments [48–57] (Table 2).

**Table 1:** Genes, their level of expression under drought condition (high, intermediate and low), their encoded proteins, log2 of the fold change (gene expression level), the nucleotide composition, ENC value and CAI value. (Additional information about the genes used in genetic transformation programs (shown with asterisk) is provided in Table 2).

Expression Level	Gene Name	Nucleotide. Length	Protein/ Function	Log_2_ of the Fold Change^1^	%A+T	%G+C	%G+C(1)	%G+C(2)	%G+C(3)	ENC	CAI
**High**	LOC_Os02g04780	1080	Uncharacterized protein/function	6.71	31.2	68.8	71.4	42.8	92.2	33.8	0.908
	LOC_Os05g39250	504	Phosphatidylethanolamine-binding protein	8.38	29.2	70.8	71.4	43.5	97.6	31.1	0.922
	LOC_Os01g50910	648	LEA protein	7.53	30.1	69.9	64.4	54.6	90.7	27.4	0.919
	LOC_Os05g33960	1815	Peptide transporter PTR2	5.03	31.4	68.7	63.5	48.1	94.4	32.2	0.918
	LOC_Os10g39920	387	Uncharacterized protein	6.01	26.6	73.4	67.4	57.4	95.3	28.1	0.926
	LOC_Os07g05940	1749	9-cis-Epoxycarotenoid dioxygenase 1, chloroplast	7.88	28.2	71.8	72	48	95.2	31.1	0.925
	LOC_Os03g60580	453	actin-Depolymerizing factor	5.89	30.9	69.1	63.6	46.4	97.4	30.3	0.946
	LOC_Os09g15670	1077	Protein phosphatase 2C	8.77	26.4	73.6	70.2	56.8	93.9	34.5	0.9
	LOC_Os11g26780	495	Dehydrin family	9.49	31.1	68.9	64.8	49.7	92.1	33.4	0.919
	LOC_Os07g24000	522	AWPM-19–like membrane family protein	6.41	31.8	68.2	64.4	46	94.3	31.2	0.934
	LOC_Os01g66120*	912	LEA protein	5.46	30.2	69.8	63.5	49.3	96.7	30.6	0.929
	LOC_Os03g60080*	951	LEA protein	6.96	32.8	67.2	61.2	48.6	91.8	32.8	0.908
	LOC_Os05g10670*	1395	NAC transcription factor	2.28	28.1	71.9	65.6	60.4	89.7	34.4	0.894
	LOC_Os03g60560*	513	NAC transcription factor	2.47	27.1	72.9	65.5	56.7	96.5	31.5	0.922
	LOC_Os12g39400*	753	Zinc finger gene family	2.45	25.2	74.8	72.9	59	92.4	30	0.895
	LOC_Os08g31580*	843	Zinc finger gene family	3.16	27.8	72.2	67.3	55.9	93.6	30.6	0.913
	LOC_Os03g20680*	1035	Zinc finger gene family	4.28	27.2	72.8	66.7	52.8	98.8	29.7	0.943
	LOC_Os05g46480*	603	AP2/ERF transcription factor	2.55	30.7	69.3	61.2	52.2	94.5	31.1	0.931
	LOC_Os03g57240*	906	C2H2 zinc finger protein	2.8	27.3	72.7	65.2	63.6	89.4	36.3	0.859
	LOC_Os05g34830*	969	NAC transcription factor	6.54	28.8	71.2	66.3	53.6	93.8	32	0.919
**Intermediate**	LOC_Os01g04840	1347	Vacuolar protein sorting-associating protein 4B	0	48.9	51.1	53.7	41.6	57.9	55.6	0.805
	LOC_Os01g04930	885	MYB family transcription factor	0	38.8	61.2	56.3	47.8	79.7	46.8	0.867
	LOC_Os01g05020	2016	Uncharacterized protein	0	43.5	56.5	54.6	36.8	78.3	46.8	0.859
	LOC_Os01g05090	657	Uncharacterized protein	0	54.9	45.1	47	44.7	43.4	50	0.750
	LOC_Os01g05100	1014	Uncharacterized protein	0	51.1	48.9	53.6	42.3	50.9	52.1	0.760
	LOC_Os01g05180	426	Uncharacterized protein	0	35.4	64.6	59.9	57.7	76.1	47.2	0.852
	LOC_Os01g05289	339	Uncharacterized protein	0	33.9	66.1	68.1	60.2	69.9	51.7	0.799
	LOC_Os01g05420	1602	Uncharacterized protein	0	47.0	53	61.8	40.8	56.4	55.9	0.789
	LOC_Os01g05440	1236	XH domain-containing protein	0	43.2	56.8	59.2	42.5	68.7	49.9	0.821
	LOC_Os01g05470	2103	Transcription factor X1	0	47.9	52.1	59.6	37.7	58.9	56	0.797
	LOC_Os01g05480	1665	Uncharacterized protein	0	44.0	56	55.3	42.7	69.9	49.4	0.832
	LOC_Os01g05490	762	Triose phosphate isomerase, cytosolic	0	50.7	49.3	57.9	41.7	48.4	50.4	0.772
	LOC_Os01g05520	321	Uncharacterized protein	0	39.6	60.4	71	52.3	57.9	48.9	0.744
	LOC_Os01g05540	1200	Uncharacterized protein	0	43.0	57	57	44.5	69.5	49.4	0.844
	LOC_Os01g05620	2970	NBS-LRR disease-resistant protein	0	57.8	42.2	50.3	33	43.1	51.1	0.733
	LOC_Os01g05744	678	Uncharacterized protein	0	44.9	46.2	40.7	38.5	59.3	53.7	0.792
	LOC_Os01g05940	4233	Receptor kinase	0	52.1	47.9	49.8	41.7	52.2	55.9	0.766
	LOC_Os01g06050	1377	Uncharacterized protein	0	49.3	50.7	55.6	48.6	47.9	56.1	0.776
	LOC_Os01g06140	924	Uncharacterized protein	0	46.4	53.6	51.6	45.5	63.6	52.6	0.809
	LOC_Os01g06320	921	MYB family transcription factor	0	44.3	55.7	53.1	44.3	69.7	50	0.837
**Low**	LOC_Os04g11060	177	Uncharacterized protein	-7.59	53.1	46.9	54.2	28.8	57.6	47	0.777
	LOC_Os11g24140	375	Plastocyanin-like domain-containing protein	-10.6	39.7	60.3	57.6	48	75.2	51.3	0.831
	LOC_Os09g19820	1173	Aminopeptidase	-5.31	58.4	41.6	49.4	35.8	39.6	51.7	0.735
	LOC_Os02g54180	399	Uncharacterized protein	-6.39	46.9	53.1	52.6	45.9	60.9	53.8	0.802
	LOC_Os07g23430	1410	Fatty acid desaturase	-4.67	37.0	63	59.6	45.5	84	40.3	0.870
	LOC_Os07g23410	1173	Fatty acid desaturase	-6.44	37.2	62.8	59.3	41.7	87.5	38.6	0.894
	LOC_Os04g11040	270	Uncharacterized protein	-6.05	44.1	55.9	57.8	50	60	42.7	0.794
	LOC_Os12g38290	231	Metallothionein	-7.68	40.3	59.7	46.8	61	71.4	47.6	0.849
	LOC_Os11g20160	1107	O-methyltransferase	-9.68	38.2	61.8	64.8	41.2	79.4	44.4	0.869
	LOC_Os11g14910	1080	NADP-dependent oxidoreductase	-8.59	45.7	54.3	59.4	38.9	64.4	53.8	0.803
	LOC_Os11g04350	1071	Cell death–associated protein	-7.52	33.3	66.7	70.6	46.5	82.9	39.3	0.876
	LOC_Os06g18960	2571	embryogenesis Transmembrane protein	--5.9	41.8	58.2	58.8	43.3	72.5	48.6	0.830
	LOC_Os01g72140	696	Glutathione S-transferase	-5.76	39.5	60.5	64.7	37.1	79.7	41.8	0.864
	LOC_Os07g04960	456	app1	-5.75	29.2	70.8	70.4	58.6	83.6	41.6	0.884
	LOC_Os11g37700	4368	Pleiotropic drug-resistant protein	-5.74	53.7	46.3	49.5	41	48.4	56.5	0.753
	LOC_Os08g37250	858	Patatin	-5.69	46.0	54	51.4	35.7	74.8	50.6	0.861
	LOC_Os03g46150	732	LTPL72—Protease Inhibitor/seed storage/LTP family protein precursor	-5.67	30.3	69.7	64.8	64.8	79.5	40.2	0.846
	LOC_Os10g02070	981	Peroxidase precursor	-5.63	46.4	53.6	54.7	40.4	65.7	50.7	0.820
	LOC_Os05g16930	2679	Protein kinase	-5.48	56.1	43.9	48.2	41.9	41.7	54.9	0.729
	LOC_Os03g05530	1125	Nodulin	-5.23	44.2	55.8	48.5	40.5	78.4	44.1	0.842

^1^ Relative expression of rice genes under drought stress. Values are shown in log_2_ of the fold changes (FC) between expression values of each gene under control and drought conditions. Genes with FC above 1 were counted as up regulated, and, below -1, down regulated.

**Table 2:** High expression genes that have been used in genetic engineering programs for enhanced stress tolerance.

Name	Gene	Protein	Observation	Reference
LOC_Os03g20680	*Os LEA3-2*	LEA protein	The transgenic rice showed significantly stronger growth performance than control under salinity or osmotic stress conditions and were able to recover after 20 days of drought stress.	[43]
LOC_Os05g46480	*Os LEA3-1*	LEA protein	Overexpression of an LEA gene in rice improved drought resistance under the field conditions without yield penalty.	[44]
LOC_Os01g66120	*SNC2*	NAC transcription factor	Overexpression of *SNAC2* improved the tolerance to PEG treatment. The transgenic plants had higher cell membrane stability and higher germination and growth rate than wild type during the cold and salt stresses respectively.	[45]
LOC_Os03g60080	*OsNAC9*	NAC transcription factor	Overexpression of *OsNAC9* gene increased rice drought tolerance.	[46]
LOC_Os12g39400	*ZFP252*	Zinc finger	Overexpression of *ZFP252* in rice increased the amount of free proline and soluble sugars enhanced rice tolerance to salt and drought stresses.	[47]
LOC_Os03g60560	*ZFP182*	Zinc finger	Overexpression of *ZFP182* significantly enhanced salt, cold and drought tolerance in transgenic rice.	[48]
LOC_Os05g10670	*OsTZF1*	Zinc finger gene family	Overexpression of *OsTZF1* in rice showed improved tolerance to high-salt and drought stresses.	[49]
LOC_Os08g31580	*OsDRAP1*	AP2/ERF transcription factor	Overexpressing OsDRAP1 transgenic plants exhibited significantly improved drought tolerance.	[50]
LOC_Os03g57240	*DST*	C2H2 zinc finger protein	A zinc finger protein, *DST*, regulated drought and salt tolerance in rice via stomatal aperture control.	[51]
LOC_Os05g34830	*OsNAC52*	NAC transcription factor	Overexpression of *OsNAC52* activated the expression of downstream genes in transgenic *Arabidopsis*, resulting in enhanced tolerance to drought stresses but not growth retardation.	[52]

### Analysis of the nucleotide composition

The program CAIcal [58] was used to calculate the AT content and GC content at first, second and the third nucleotide positions, (AT1, AT2, AT3) and (GC1, GC2, GC3), respectively.

### Relative Synonymous Codon Usage (RSCU) analysis

RSCU was calculated following Hastings and Emerson [59], as the ratio of the observed frequency of a codon to the expected frequency of the same codon within a synonymous codon group (with no bias) in the entire coding sequence of the gene concerned. RSCU can be equal to one when there is no bias, more than one when there is preference to use that specific codon and less than one when the codon is underused [60]. RSCU values can range from zero where the specific codon is not used at all to 2, 3, 4 and 6 when only one codon is used for encoding amino acids with 2, 3, 4 and 6 synonymous codons, respectively. An RSCU value >1 for each codon shows that this codon is preferred. Higher RSCU value indicates the presence of higher CUB. The RSCU of 60 genes associated with drought stress in rice were calculated using ACUA software [61] and the program CAIcal [58], excluding the stop codons and the two amino acids which are encoded by a single codon (Trp and Met).

### ENC analysis

Effective number of codon usage (ENC) refers to the number of unique codons found in a gene. This value can be in a range of 20 (where there is an extreme bias towards the use of only one codon for each amino acid) to 61 (representing the use of all synonymous codons). If there are fewer than 60 synonymous codons used in a particular gene, then CUB may be present (considering the fact that Trp has only one codon). We used CAIcal to calculate ENC.

### Codon Adaptation Index (CAI) analysis

CAI is another widely used method for evaluation of CUB. It measures the similarity between the frequency of synonymous codons used by a gene and that of a reference set [58]. The range of the CAI value is between 0 and 1. The rice codon usage from the Kazusa database [62] was used as a reference set. The program CAIcal [58] was used for CAI calculation.

### Searching for tRFs data under drought stress

Data on tRFs under drought stress in rice were obtained from the PtRFdb (http://223.31.159.8/PtRFdb/plant.php?txt=Oryza%20sativa&bs=1&type=all) [63] and their frequency was compared in the control and drought stress condition in different stages of plant growth. Codons related to each tRF were also compared with the rice preferred codons under drought stress.

## Results

### Base composition analysis

**Fig 1:**
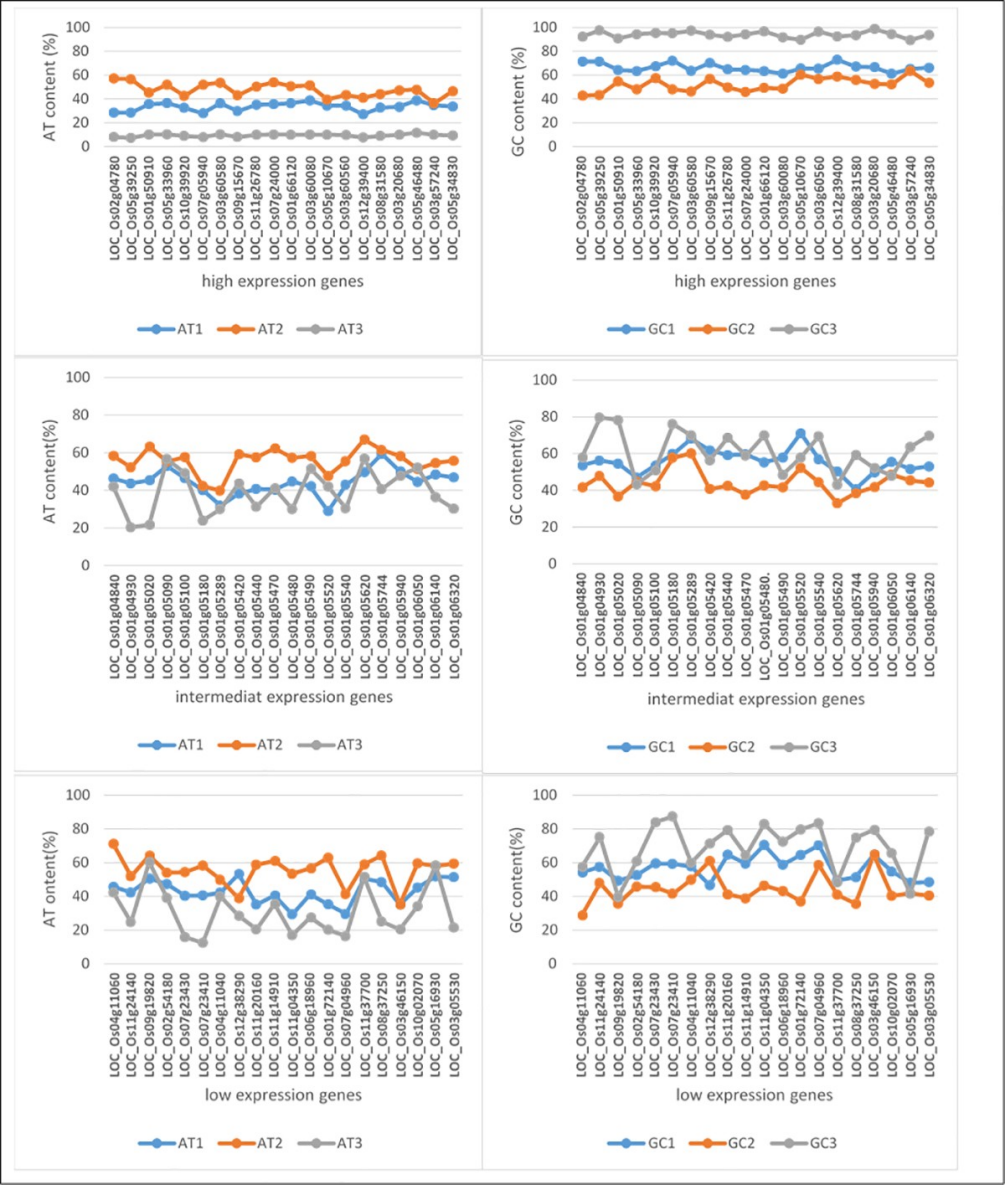
The nucleotide composition of three gene expression categories under drought stress (high, intermediate and low expression) in 60 selected rice genes.

In the present study, GC content was higher than AT content in all three gene-expression categories (high, intermediate and low-expression genes), as is expected for the rice genome. The means of GC content in categories 1, 2 and 3 were 70.9%, 56.95% and 54.2%, respectively. The results showed that the GC content at the three-codon positions was noticeably different. G or C at position three of each codon (GC3) was higher than that at position one (GC1), and it was lowest at position two (GC2) among most of the studied genes. The means of GC3 content in categories 1, 2 and 3 were 94%, 69.3% and 56.2%, respectively (Table 1, Fig 1).

### ENC analysis

The CUB for each gene was also calculated using ENC. The ENC value of the genes of the first group was between 27 and 34, the second group was between 38 and 56 and the third group was between 46 and 56 (Table 1).

### Analysis of CAI

The range of the CAI value in the first category was between 0.859 and 0.946; in the second and third categories were between 0.733 and 0.867 and between 0.729 and 0.894, respectively (Table 1). High expression genes showed higher CAI value as compared to the other categories.

### RSCU analysis

**Fig 2:**
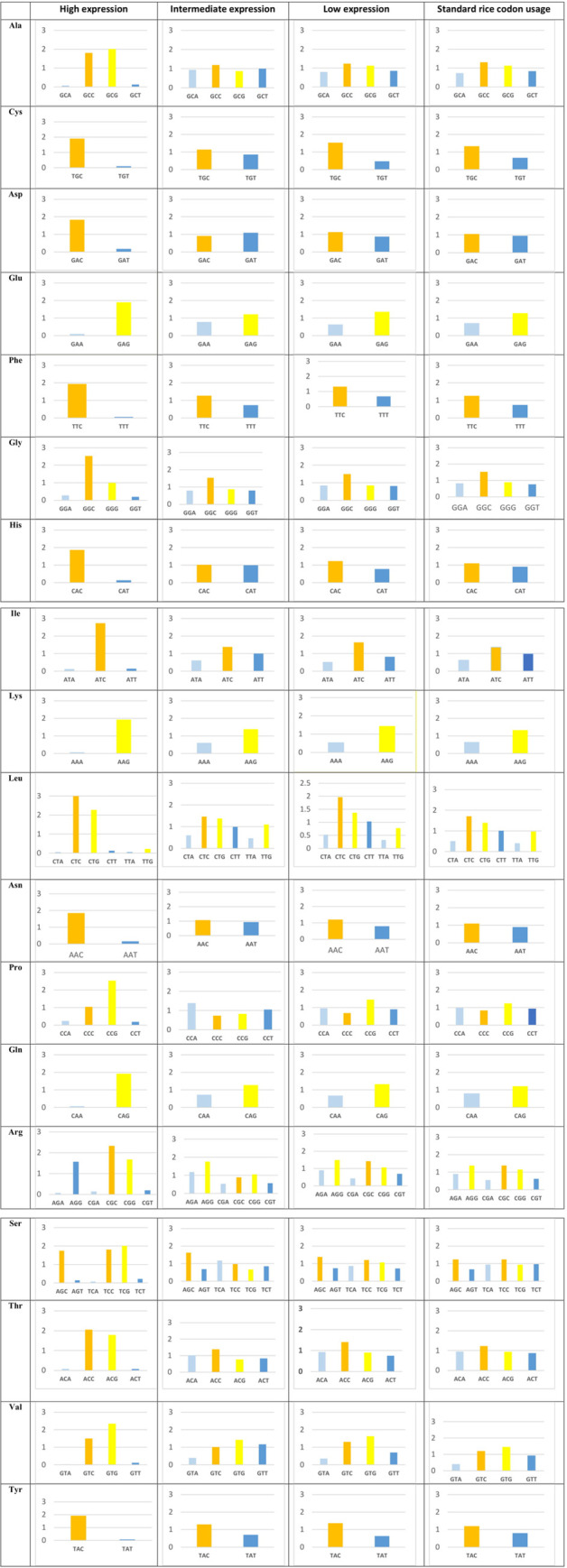
RSCU values of each codon/amino acid across 20 genes in each high, intermediate and low gene expression categories in rice.

The comparative study among the RSCU values of the studied genes in three expression categories under drought stress with that in general pattern of rice genes reveals the existence of CUB. In other words, when genes with different expression levels under drought in rice are compared with the standard rice codon usage table, a change in preferred codon of some amino acids of high expression genes and low expression genes under stress is observed. For example, alanine and serine are encoded by the most preferred codon GCG and TCG instead of rice’s most widely used codons GCC and TCC, respectively, in the high expression genes category. The third codon position changed to the nucleotide G in this category. Aspartic acid and proline are encoded by the most preferred codon GAT and CCA instead of rice standard codons for these two amino acids: GAC and CCG, respectively, in the intermediate expression genes category. The third codon position changed to the nucleotides A and T in this category (Fig 2).

**Fig 3:**
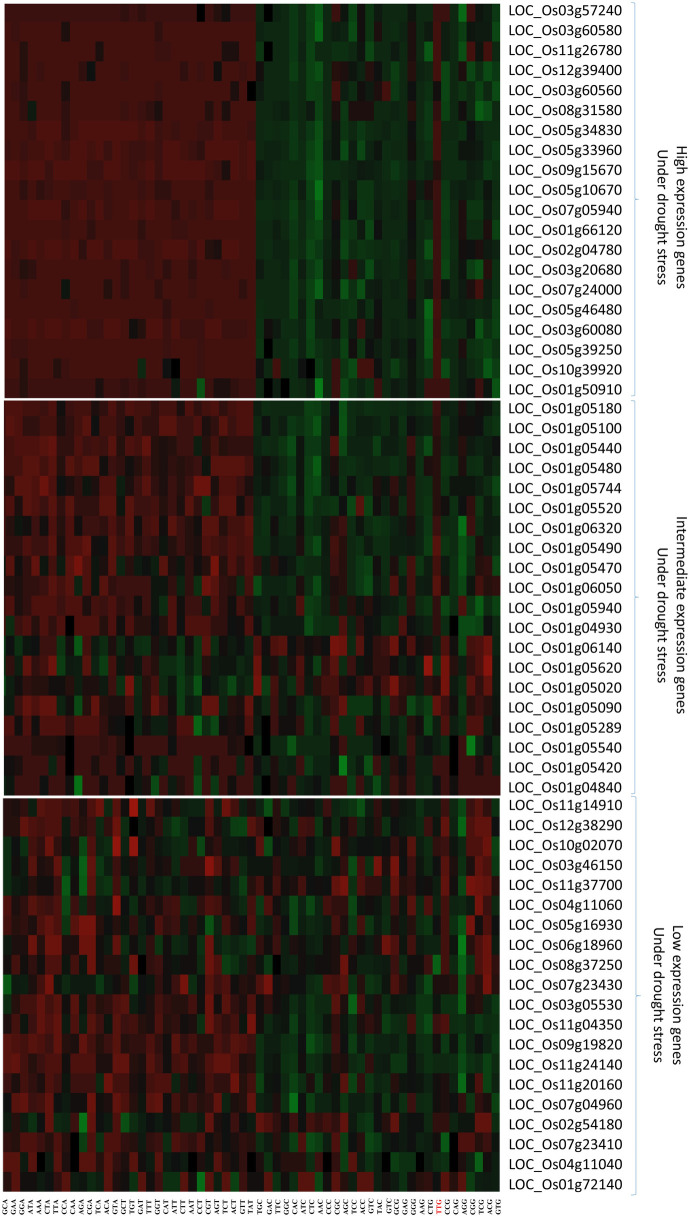
The heat-plots of codon usage profiles for high, intermediate and low gene expression categories under drought stress in rice based on RSCU index. The green color represents high propensity for using the specified codon, whereas the red color shows lower propensity and the black color shows intermediate tendency. TTG codon (marked with red) was not preferred for leucine in high expression genes under drought stress.

The heat-plot of different gene categories based on the RSCU index is shown in Fig 3. This plot separates distinctly the high expression genes under drought conditions from other categories so that the colors were divided into two distinct groups (Fig 3). These data may indicate that genes with higher expression under drought stress have acquired their own preferred codons in the course of evolution. Also, the heat-plots showed that G or C end codons are highly preferred in this set of genes for most amino acid codons, but the TTG codon was not preferred for leucine in high expression genes under drought stress.

### Average RSCU (GC/AT): A new index for estimation of CUB

**Fig 4:**
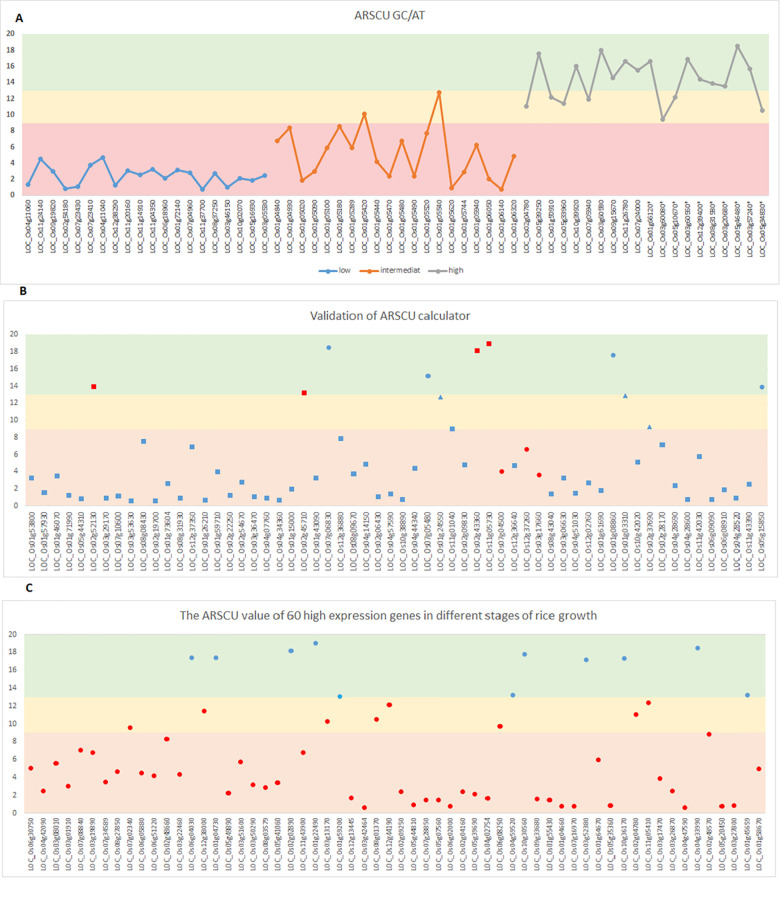
(A) The ARSCU GC/AT value of high, intermediate and low expression gene categories in rice; (B) Validation of ARSCU-Calculator using 60 rice drought stress related genes, randomly selected and calculated by the program. (C) The ARSCU value of 60 high expressing non drought related genes. Genes with ARSCU above 13 are estimated as high expression genes under drought conditions) green area. (ARSCU between 9 to 13 are estimated as genes with high or intermediate expression (yellow area). ARSCU less than 9 are estimated as genes with low expression or intermediate expression (pink area). Blue and red shapes: correct and wrong prediction, respectively. High, intermediate and low-expression genes are indicated with solid circle, triangle and square, respectively.

After calculating the RSCU index for each codon per gene, it was observed that the ratio of RSCU of codons with GC end to codons with AT end in each amino acid was significantly higher in high expression genes than that in the other categories. Even in some high expression genes, the RSCU of codons with AT end was zero. Hence, an RSCU-based index was defined and called ARSCU. It measures the average ratio of RSCUs with GC- to AT-end codons for all amino acids in one gene, providing only one value for each gene. Our findings indicate that ARSCU could significantly separate highly expressed genes under drought from the others. In this study, the ARSCU (GC/AT) index ranged between 9.5 to 18.5, 0.7 to 8.5, and 0.7 to 4.5 for high, intermediate and low expression genes, respectively (Fig 4A).

ARSCU was calculated as:

$$ARSCU=\left(\frac{\sum_{aa:1}^{aa18}a/b}{18}\right)$$

Where aa is amino acid, a is RSCU of GC end codons and b is RSCU of AT end codons (any a and b with a value of zero is arbitrarily assigned a value of 0.1).

### ARSCU- calculator

A program was developed in an Excel file for calculating the ARSCU index (S1 Table). Input data for this program is the RSCU index of all amino acid codons in each gene. This program calculates only one ARSCU (GC/AT) for each gene (whilst every gene has 59 RSCU values). Since RSCU may be zero for some codons, any RSCU with a value of zero is arbitrarily assigned a value of 0.1.

Based on our findings, ARSCU values can be used for discrimination of genes with different expressions under drought conditions, as follows:

- Genes with ARSCU above 13 (arbitrary threshold) are predicted to be high expression genes under drought conditions;
- Genes with ARSCU ranging from 9 to 13 are predicted to be genes with high or intermediate expression;
- Genes with ARSCU numbers less than 9 are predicted to be genes with low or intermediate expression.

### Validation of discriminating power of ARSCU

For validation of discriminating power and accuracy of the ARSCU, 60 other rice genes were randomly selected from 43233 genes studied for their response to drought stress [44] and their ARSCU were calculated by the program (S2 Table). The results were correlated with the expression datafrom the published meta-analysis [44]. ARSCU calculator was able to predict genes with different expression level under drought stress in rice with an accuracy of 88.3%) Fig 4B).

In order to compare the codon usage pattern of different highly expressing genes unrelated to drought with that of drought responsive high expression genes, third set of 60 other highly expressed genes not related to drought [64,65] were used. The ARSCU value for these genes was also calculated by the program) S3 Table). Only 31.65% of the genes (18.33% plus 13.33% were correctly categorized as high and intermediate expressing genes, respectively) similar to those obtained for drought responsive genes (Fig 4C). ARSCU as an index worked for identification of drought related genes (with 88% accuracy) compared to only 32% accuracy on non-drought related genes.

### tRFs under drought stress

Results of our search for tRFs under drought stress and control condition in rice, showed that:

1. In most cases, no tRF has been reported for the preferred codon under drought stress, which means the tRNA remains for longer time and participates in translation.
2. In few cases, tRF has been reported for tRNA related to preferred codon under drought stress. However, even in these cases the frequency of this tRF has decreased under drought stress compared with its frequency under normal condition.
3. Only in one case (Ala), tRF related to preferred codon has increased under drought stress. tRNA expression data is required in order to explain this observation. One mechanism may be over expression of the preferred tRNA in such amounts that even its break down to its tRF does not affect the over-expression of the gene in concern (Table 3).

**Table 3:** Frequency of tRFs under control and drought stress conditions in rice.

Amino Acid	Anticodon*	frequency of tRFs under control condition	frequency of tRFs under drought stress	preferred codon**	tRF for preferred codon under drought
**Cyc**	ACA	9944	8089	TGC	No
**Glu**	CTC	506	0	GAG	Yes, but decreased under drought
**Gly**	CCC	33	11	GGC	No
**His**	GTG	33	17	CAC	Yes, but decreased under drought
**Leu**	AAG	396	655	CTC	No
**Met**	CAG	340	178	None	No preferred codon-
**Asn**	GTT	97	69	AAC	Yes, but decreased under drought
**Arg**	ACG	0	17	CGC	No
**Ser**	GCT	234	244	TCG	No
**Val**	GAC	206	220	GTG	No
**Tyr**	GTA	27	0	TAC	Yes, but decreased under drought
**Ala**	CGC	1377	1947	GCG	Yes
**Asp, Phe, Ile, lys, Pro, Gln, Thr, Trp**	-	tRF not reported	tRF not reported	-	No

*Anticodon of the type of tRNA that breaks into tRF

**Preferred codons of high expression genes under drought stress

## Discussion

Drought tolerance in rice is governed by many genes with huge environmental interaction, with low heritability, and thus are difficult to study [66]. Development of GM plants with enhanced tolerance to drought is an important challenge in rice biotechnology research. For this purpose, further investigation of the mechanisms, pathways and genes involved in response to abiotic stress is essential. Expression of genes belonging to diverse functional and regulatory groups, such as transcription factors, protein kinases, and phosphatases, are influenced by abiotic stress conditions [67]. In addition to environmental conditions, gene expression levels correlate with multiple aspects of gene sequence and structure including SCU [18]. Here, we report the existence of strong correlation between SCU and gene expression levels under drought stress in rice which has not previously been reported.

### Correlation between GC content and higher expression of rice genes under drought

Our analysis of codon usage patterns of studied genes in different expression categories under drought stress shows that most of the genes in the three categories have more GC content as expected from rice genes. In this study, GC percent analysis was able to separate 60 selected genes in rice into two major classes: 1) the high expression genes that were also high in GC and 2) the other genes with lower GC content. Higher GC content in rice genome has been reported with no particular attention paid for presence or absence of differences among genes with different levels of expression under drought [68]. Wang and Hickey [69] showed that the means of GC content of 14005 rice-coding sequences was 57.8%. Also they separated the 14,005 rice genes into two classes: High (67.4%) and Low GC genes (50.1%). The means of the high GC rice genes and low GC rice genes’ GC3 were 80.4% and 52.7%, respectively in their study. Also, Yi et al. [33] showed higher expression is associated with higher GC3 in three closely-related species of the genus *Misgurnus*. Our results further confirm the previous reports in the field. However, the novelty of our findings may be the existence of a correlation between genes with different levels of expression under drought condition and their GC content. We showed that genes with high expression under drought conditions had overall 70.9% GC content. Furthermore, we showed that on average 94% of the high expressing genes contained a G or C at third position of their codons. Therefore, we have demonstrated that high expression genes under drought are more dependent on high GC percentages, especially in the third nucleotide position.

### Correlation between SCU and higher expression of rice genes under drought

As we expected, the range of the CAI value was higher in the high expression genes compared with other categories. In addition, the ENC value for the high expression genes was less than 36, which is an indication of the presence of a relatively strong CUB. This value for the second and third categories of genes was above 38 and 46, respectively. Sharp et al. [60] reported that, highly expressed genes generally show a tendency of preferentially using a limited number of codons. Also, Liu et al. [70] showed a positive correlation between ENC value and gene expression level in the rice plant. In their report, genes with ENC ≤30 and ENC ≥55 were correlated with high and low expression genes, respectively.

Alanine and serine in the high expression genes under drought stress preferred the GCG and TCG codons, respectively, whereas according to the common rice codon usage table, the preferred codons for alanine and serine were GCC and TCC, respectively. Our results about those amino acids encoded by six codons (leucine, arginine and serine) showed that among the C- or G-ending codons, using G or C in the first and second positions is also more preferred. For example, for the case of leucine, the use of the TTG codon, although ending with a G, is not preferred and the CTC and CTG codons are more preferred. Therefore, using TTG is not recommended for codon optimization for enhanced expression of the concerned gene under drought. Intermediate and low expression genes under drought stress end with A or T codons in some cases. This may further indicate the effect of more efficient codons on higher expression of genes under drought stress. For example, proline and aspartic acid preference the GAT and CCA codons in the intermediate expression genes under drought stress, whereas the preferred rice codon for these two amino acids is GAC and CCG that end with a G or C. Therefore, the use of A and T end codons for these amino acids has reduced the expression of these genes under drought stress. A number of studies of eukaryotes and prokaryotes have showed that groups or families of genes involved in stress responses systematically overuse and underuse specific “non-optimal” synonymous codons. Chionh et al. [71] showed the 48 genes in the DosR regulon in *Mycobacterium bovis* BCG, which are essential for survival under hypoxic stress, are enriched in G- and C-ending codons. Others showed that hypoxic stress increased the translation of proteins from genes enriched with non-optimal codons ACG (Thr), CTA (Leu), GCG (Ala) and GGA (Gly), whereas their synonymous partners ACC (Thr), CTT (Leu), GCT (Ala) and GGT (Gly) were all overrepresented in downregulated proteins in hypoxia [72]. High-expression genes under drought in rice use G/C-end codons more frequently. Therefore, we created a different codon usage table for these genes compared to the standard rice codon usage table (S4 Table).

Correlation of CUB with gene expression levels for different species including: *Caenorhabditis*, *Drosophila*, *Arabidopsis* [20], *Tribolium castaneum* [18], arthropods [32,72], *Paeonia lactiflora* [73] *and Populus tremula* [74] has been shown by several authors. Selection favors specific codons promoting the efficient and accurate translation of genes that are expressed at high levels.

### ARSCU as a single value for prediction of rice genes expression under drought

Previous reports have used the RSCU number for different cases, but this number is calculated for the codons of each amino acid in the gene. In this report, we introduced the ARSCU GC/AT as a single value/index for each gene with the potential to predict gene expression under drought stress. In addition, we developed a simple program in an Excel file for calculating the ARSCU index (ARSCU calculator). It is expected that using this program, we will be able to predict high expression genes in high throughput data with relatively high confidence. This program was developed based on calculations made on a set of genes expressed under drought stress conditions in the rice genome. We validated 1) the discriminating power of ARSCU and 2) its specificity for higher expressing genes under drought. This index was not able to predict highly expressed non-drought related genes. These findings may reflect the different codon usage pattern of the genes under drought stress compared with other highly expressed genes in rice. In other words, the observed phenomenon (strong correlation between ARSCU and high expressing genes) is restricted to higher expressing genes under drought stress and not every high expressing genes in rice.

It remains unclear if ARSCU will also be useful for prediction of gene expression in other plant species under drought or even other stressed conditions. We are currently attempting validation of this program for prediction of gene expression under drought for other cereals. It may require a different threshold definition than the one reported by this index (ARSCU) in this article. There is room for improvement of this program to be suitable for use with the codon usage features of other plant species. The prediction made by this program potentially indicates the extent to which such a codon composition can be expressed under drought. It is, however, clear that in addition to ORF, regulatory elements in particular the promoter and transcription factors affect gene expression and that these variables were not considered in this program. It is therefore expected that even in rice, genes with high ARSCUs may exhibit low expression under drought. The high ARSCUs in such genes only indicate their codon potential for high expression under stressed conditions. In the case of such genes, alteration of regulatory elements may materialize their expression potential.

### Possible role of tRNA post transcriptional modification and tRFs justifying the correlation between CUB and high expressing genes under drought

We observed different codon usage pattern in high expression genes under drought stress in our research. This observation termed Modification Tunable Transcripts (MoTTs) was also reported as a model combining genome-wide CUB analytics and gene expression studies. This model implies that modification of tRNA drives the translational regulation of critical response proteins whose transcripts display a distinct codon bias [75].

Average transcripts not exhibiting CUB, do not need specific tRNA modifications, as they are efficiently translated under all conditions. In contrast, the translation of MoTTs in cells lacking tRNA specific modifications is severely reduced [37].

The number of genes that encode tRNA modification enzymes are far more abundant than the tRNA coding genes, which further highlights the importance of tRNA modification [76,77]. It has been long postulated that modified nucleosides on tRNA molecules may function as “biosensor” for environmental and physiological changes [78,79] as a fast module to regulate gene expression at translational level. In agreement with this hypothesis, the abundance of tRNA modified nucleosides do change in response to various stresses [34]. Stress-induced degradation of tRNA has been reported by several investigators [80,81].

We speculate that those transcripts that are more efficiently expressed due to the presence of CUB during the course of stress, can be more efficiently used by translational machinery to express proteins due to the presence of specific modifications. However, this require more direct evidence to draw a final conclusion.

Methylation sensitive degradation of tRNA as another potential underlying mechanism for these observations has also the merit to look at. Wang et al. [82] investigated the mechanism of changes in the composition and abundance of tRNA-modified nucleosides in response to drought, salt and cold stress, as well as in different tissues during the whole growth season in two model plants–*O*. *sativa* and *Arabidopsis thaliana*. They identified 22 and 20 candidate genes for methyl-transferases in rice and Arabidopsis, respectively. Based on bioinformatics analysis, nucleoside abundance assessments and gene expression profiling, they found four methylated nucleosides (Am, Cm, m^1^A and m^7^G) that are critical for stress response in rice and Arabidopsis.

However, the relationship of these modifications with CUB is not yet known. On the other hand, several articles have discussed tRNA modification of the animal species and its association with codon usage. Chan et al [74] showed a model for translational control mechanism for survival under oxidative stress in yeast. Exposure to H_2_O_2_ leads to an elevation in the level of m^5^C at the wobble position of the leucine tRNA for translating the codon UUG on mRNA. This modification in tRNA enhanced the translation of the UUG-enriched RPL22A mRNA relative to its paralog RPL22B and leads to changes in ribosome composition in their study. This reprogramming of tRNA and ribosomes ultimately causes selective translation of proteins from genes enriched with the codon TTG. More comprehensive studies are needed to better correlate the expression of genes with specific codon preference under drought stress in rice with tRNA modifications.

**Fig 5:**
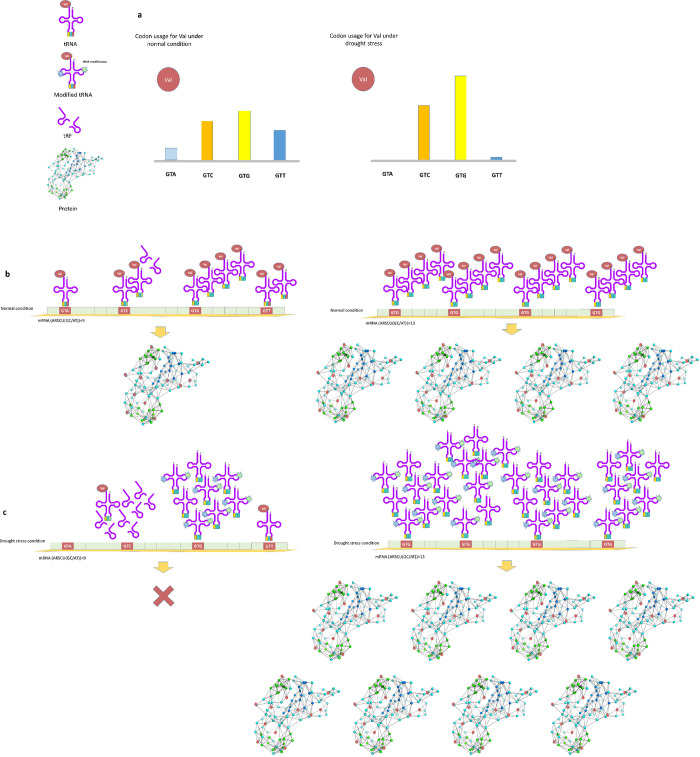
Proposed model justifying codon optimization in genetic engineering programs based of the observed CUB, codon characteristics, tRNA modification and tRFs under drought stress in rice. a) The codon usage difference under normal and drought condition for amino acid Valine. b) A segment of mRNA is shown under normal condition, before (left) and after codon optimization (right) symbolizing a gene. Under normal conditions, both genes (before and after codon optimization) are expressed. However, the codon-optimized gene is more expressed [83]. c) Native genes are minimally expressed or no expressed under stress (left) compared to the optimized genes (right). Under stress conditions (C), tRNA modifying enzymes mark a series of tRNAs that carry preferred codons leading to increased translation of codon optimized gene (right). In the case of amino acid Valine, the tRNA associated with GTC codon is broken under stress in the form of tRFs. It makes limited access to the codon for translation.

The fact that in most cases, no tRF has been reported for the preferred codon under drought stress, may support our hypothesis that during the course of evolution plants with preferred codons for which the tRNA does not break down into tRF gained selective advantage under drought condition. This may be the result of preservation of already existing such preferred codon or even occurrence of the mutation creating the preferred codon. This may be considered for further studies with the objective of identifying the underlying biological mechanism of CUB observed for high expression genes under drought. Based on these observations we propose an illustrated model based on codon characteristics, tRNA modification and tRFs under drought stress in rice (Fig 5).

In summary, the results of this paper suggests that codon optimization may play more significant role for improved gene expression under drought. Therefore, it is suggested to be considered in genetic engineering programs. It is expected that drought tolerant rice will be available from our ongoing projects on engineering rice for enhanced drought tolerance using several different strategies. The genes used in our projects have been optimized based on the findings reported in the current study.

## Supporting information

S1 Table ARSCU- calculator. (XLSX)

S2 Table Validation of ARSCU-calculator using 60 rice drought stress related genes. (XLSX)

S3 Table The ARSCU value of 60 high expression genes in different stages of rice growth. (XLSX)

S4 Table Rice codon usage table compare with rice high expression genes under drought stress. (XLSX)
